# Large-Scale Non-Targeted Metabolomics Reveals Antioxidant, Nutraceutical and Therapeutic Potentials of Sorghum

**DOI:** 10.3390/antiox10101511

**Published:** 2021-09-23

**Authors:** Ajay Prasanth Ramalingam, Williams Mohanavel, Ameena Premnath, Raveendran Muthurajan, P. V. Vara Prasad, Ramasamy Perumal

**Affiliations:** 1 Department of Plant Biotechnology, Tamil Nadu Agricultural University, Coimbatore 641003, Tamil Nadu, India; realajax97@gmail.com (A.P.R.); greenwilliamsmohanram@gmail.com (W.M.); ameenaprem@gmail.com (A.P.); 2 Department of Agronomy, Kansas State University, Manhattan, KS 66506, USA; vara@ksu.edu; 3 Agricultural Research Center, Kansas State University, Hays, KS 67601, USA

**Keywords:** sorghum, metabolomics, antioxidants, phytosterols, flavonoids, biofortification

## Abstract

Sorghum is one of the most important food and feed cereal crops and has been gaining industrial importance in recent years for its biofuel, nutraceutical and antioxidant values. A genetic profile variation study was undertaken for the accumulation of phytochemicals in 61 diverse sorghum accessions differing in their growth habitat and grain color through non-targeted Gas Chromatography–Mass Spectrometry (GC-MS/MS) analysis. Mass Spectrometry–Data Independent AnaLysis (MS-DIAL) and MetaboAnalyst identified 221 metabolites belonging to 27 different phytochemicals. Tropical and temperate sorghums were distinct in their metabolic profiles with minimum overlaps, and 51 different metabolites were crucial in differentiating the two groups. Temperate sorghums had the ability to accumulate more of phenolic acids, phytosterols, flavonoids, carotenoids, and tropical sorghums for stress-related amino acids, sugars and fatty acids. Grain-color-based Partial Least Square–Discriminant Analysis (PLS-DA) analysis identified 94 Variable Importance in Projections (VIP) metabolites containing majority of flavonoids, phenylpropanoids and phytosterols. This study identified two sorghum lines (IS 7748 and IS 14861) with rich amounts of antioxidants (catechins and epicatechins) belonging to the group of condensed tannins that otherwise do not accumulate commonly in sorghum. Out of 13 metabolic pathways identified, flavonoid biosynthesis showed the highest expression. This study provided new opportunities for developing biofortified sorghum with enhanced nutraceutical and therapeutics through molecular breeding and metabolic engineering.

## 1. Introduction

Sorghum (*Sorghum bicolor* (L.) Moench) is the fifth important cereal crop globally after rice (*Oryza sativa* L.), wheat (*Triticum aestivum* L.), maize (*Zea mays* L.) and barley (*Hordeum vulgare* L.), with 40.1 million ha under cultivation and 57.9 million metric tonnes of grain production [1,2]. Sorghum is highly adapted to marginal and stressful environments and remains the crop of choice in semi-arid regions [2]. Sorghum has diverse applications, such as grain as food and feed, fodder and forage sorghum for pasture and hay in livestock feed, broomcorn sorghum for making brooms and sweet sorghum for biofuel and syrup production. Sorghum is used for industrial applications such as biscuit industries, therapeutics, synthesis of organic compounds, and utility items. A declining trend in sorghum consumption in recent years is attributed to increasing urbanization and a lack of diversified value-added products in sorghum. Under a changing climatic scenario, sorghum can be considered as a better alternative to rice, wheat or maize due to its high adaptability to marginal environments [3,4].

Changing human lifestyles and increased frequency due to the occurrence of new diseases, namely, obesity, diabetes and cardio vascular diseases, requires a change in the human diet [5]. In this context, sorghum has been rated as a valuable and economically functional food due to the possession of unique bioactive compounds, namely, flavonoids, phytosterols and polyphenols, exhibiting immense health benefits, including anti-cancer and antioxidant potentials [6,7,8,9,10,11]. Sorghum bran is a rich source of various phytochemicals and antioxidants [12,13]. Antioxidant activities can be attributed to their phenolic compounds [6,14,15,16,17].

Sorghum grains are used to produce functional snacks [18,19,20,21], beverages [22,23,24], food colorants [25], meat preservatives [26] and animal feed [27]. Therapeutic potentials of sorghum indicated its suitability to patients with diabetes and cardiovascular problems [28,29]. Encapsulated sorghum tannins rich in antioxidants were found to reduce the rate of gastric digestion and found to exhibit anti-hyperglycaemic effects [30,31]. Non-targeted metabolomics in the kernels of diverse rice and maize genotypes using GC-MS and UHPLC-MS/MS revealed the genotypic differences in the accumulation of bioactive compounds [32,33]. Tugizimana et al. (2019) [34] conducted metabolomic analysis of disease responsiveness in three different sorghum accessions and identified key pathways reprogrammed during disease progression. Turner et al. (2016) [35] analyzed the metabolome profiles of 11 sorghum lines and revealed that accumulation of primary and secondary metabolites are tightly related to photosynthesis and biomass accumulation. In another study, 217 metabolites differentiating white, red and purple sorghum grains were identified [36]. Brewing sorghum accessions exhibited higher levels of antioxidant properties due to the possession of phenolic acids and proanthocyanidins [8]. Normally, most sorghums contain common antioxidants, namely, phenoloic acids, flavonoids and anthocyanins, but accumulation of effective antioxidant “condensed tannins” is rarely reported [37]. In the present study, a large-scale non-targeted metabolomic analysis was carried out in a set of 61 sorghum accessions differing in grain color and geographical origin. Results of this study unravelled the metabolome complexity and nutraceutical, therapeutic and antioxidant potential of sorghum grains. Outcomes of this study provide a pathway for dissecting the genetic control of metabolite accumulation in sorghum, which will in turn accelerate the development of biofortified sorghum varieties through metabolic engineering and molecular breeding.

## 2. Materials and Methods

### 2.1. Genetic Materials

**Figure 1:**
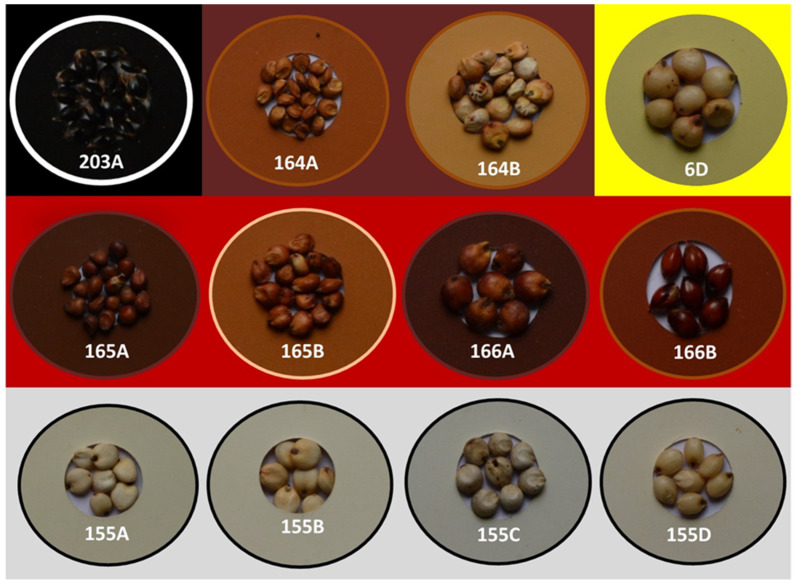
Assigning grain color to the diverse sorghum accessions using Royal Horticulture Society (RHS) color chart.

Present study was carried out in a subset of 61 sorghum accessions exhibiting wider geographic and genetic diversity with varied grain color obtained from National Bureau of Plant Genetic Resources (NBPGR), New Delhi, India (Supplementary Table S1 and Figure 1). The study materials were representative of five different grain color categories: black (2), brown (21), red (17), white (20) and yellow (1). All the accessions (10 plants each accession) were evaluated in a randomized block design during *Rabi* season (October to March) 2019 at Agricultural Research Station, Kovilpatti, India (Latitude 9.17′ N, Longitude 77.88′ E) [38] under dryland condition. Three panicles were selfed prior to flowering to avoid outcrossing. Seeds from the selfed panicles were used for color grading and metabolomics studies.

### 2.2. Grading of Grain Color

Grains of all the accessions were graded using Royal Horticulture Society (RHS) color chart [39]. Grain color, namely, black, brown, yellow, red or white, was assigned by matching with the sorghum descriptor color grade following the standards of the International Board for Plant Genetic Resources, Rome, Italy [40] (Figure 1).

### 2.3. Extraction of Secondary Metabolites and Gas Chromatography-Mass Spectrometry Analysis

Secondary metabolites were extracted from the grains of 61 accessions using Soxhlet extraction procedure [41]. Ten grams of grain samples were powdered using mixer grinder, packed with countryman filter paper and kept in the extraction chamber. Boiling flask with 300 mL sonicated 100% methanol (High Performance Liquid Chromatography, HPLC) grade was attached with the extraction chamber provided with a condenser above it. Extraction was started by heating the flask to an initial temperature of 40 °C for 10 min followed by heating at 60 °C for 10 min and finally increased to 80 °C till the completion of five cycles. The collected methanol extract was collected in a conical flask and air-dried until the volume of extract reached 5 mL. Gas chromatography-MS/MS analysis was performed using Perkin Elmer 680 GC (Perkin Elmer Inc, Akron, OH, USA) instrument coupled with AxION iQT’s MASS IQ software for data acquisition with DB-5 MS Capillary Standard non-polar column (30 Mts, ID: 0.25 mm, Film: 0.25 IM, (Perkin Elmer Inc, Akron, OH, USA)). One mL of methanolic extract of the sample was injected into the column using helium as the carrier gas. GC-MS/MS analysis was performed with mass range scan of 50–1000 *m*/*z*, 70 eV was applied for fragmentation and precursor ions were isolated with an isolation window of 10 *m*/*z*. Raw mass spectra obtained were converted to .abf format using ABF converter <www.reifycs.com/AbfConverter/> (accessed on 19 November 2020) for further analysis.

### 2.4. Data Processing and Peak Annotation

MS-DIAL (mass spectrometry-data independent analysis) was used for processing the data in .abf format [42], from which MS/MS was performed in data-dependent mode (Supplementary Figure S1). Based on the grain color, samples were grouped into four, namely, brown (21), red (17), white (20) and others (black (2); yellow (1). The processed mass spectra data consisting of peak masses and its area intensities were generated using default MS-DIAL parameters. This included MS1 and MS2 being centroid; ion mode-positive; mass range 0–1000 *m*/*z*; retention time range 0–30 min; mass tolerance of 0.25 *m*/*z*; retention time tolerance (5 s); minimum peak width and height 5 and 1000; deconvolution parameters (sigma value—0.5); data filtering (inter-quantile range); normalization (normalization by sum); data transformation (log); and data scaling (mean centering). Annotation was done on MS-DIAL using publicly available libraries in “.msp format” from MassBank of North America (MoNA), including Massbank and HMDB <http://www.hmdb.ca/> (accessed on 19 November 2020), by comparing the processed mass spectra data against the libraries with 80% identification score cut off [43].

### 2.5. Statistical Analysis

The processed data were used to perform statistical analysis using the web platform ‘MetaboAnalyst 5.0’ www.metaboanalyst.ca (accessed on 19 November 2020) [44] with the missing values being replaced by 1/5 of minimum positive values of their corresponding variables. Analyses, including univariate model like one way analysis of variance (ANOVA), multivariate models such as principal component analysis (PCA), partial least squares discriminant analyses (PLS-DA) and hierarchical clustering were performed for understanding metabolite variation and identifying significant metabolites. Fold change analysis was performed by keeping a threshold value of two for determining up-regulated and down-regulated metabolites between colored and white grain groups, followed by mapping the regulated metabolites in sorghum secondary metabolism pathway using MapMan <https://mapman.gabipd.org/> (accessed on 19 November 2020) [45].

### 2.6. Pathway Mapping

Significant metabolites exhibiting contrasting differences between different color groups were mapped onto metabolic pathways using MetaboAnalyst 5.0 [44,46]. Mummichog algorithm was used to predict the pathway analysis of the processed data using Kyoto Encyclopaedia of Genes and Genomes (KEGG) database <www.genome.jp/kegg/pathway.html> (accessed on 19 November 2020) [47]. False discovery ratio for the pathway analysis was set to ≤0.05.

## 3. Results

### 3.1. Genetic Variation for Grain Color

Based on the descriptors and color chart of Royal Horticulture Society, 61 sorghum accessions were classified into black, brown, red, yellow and white grains (Supplementary Table S1; Figure 1). Adequate care was taken to include sorghum accessions representing all the major grain color groups, namely, black (203A; 2 accessions), brown (164A and 164B; 21 accessions), red (165A, 165B, 166A and 166B; 17 accessions), white (155A, 155B, 155C and 155D; 20 accessions) and yellow (6D; 1 accession).

### 3.2. Metabolic Profile

**Figure 2:**
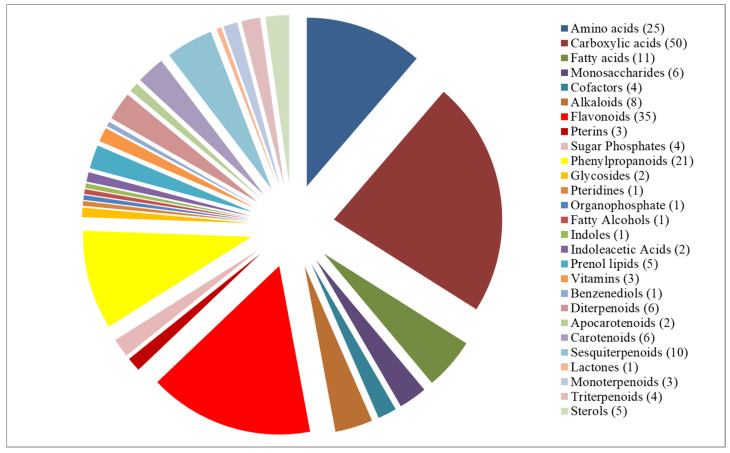
Classification of 221 sorghum grain metabolites into metabolite classes. Number in parentheses indicates number of metabolites mapped against each class.

Mass spectrometry data analysis identified a total of 221 known compounds (Supplementary Figure S2; Supplementary Table S2), including varied classes of primary and secondary metabolites (Figure 2) mapping onto 27 sub-pathways listed in KEGG database (Table 1). These 221 metabolites belonged to diverse categories of metabolism (Figure 2). Among the metabolites, predominant ones were in the order of carboxylic acids (50), flavonoids (35), amino acids (25), phenylpropanoids (21) and sesquiterpenoids (10).

**Figure 3:**
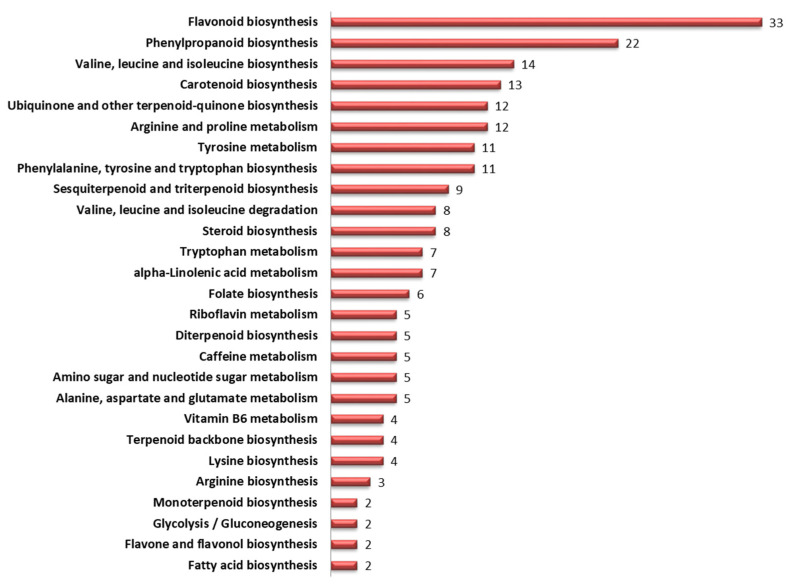
Mapping of sorghum grain metabolites onto KEGG (Kyoto Encyclopaedia of Genes and Genomes) metabolic pathways. Values indicates the number of metabolites mapped against various specific pathways.

Many of the metabolites were mapped onto therapeutically important pathways such as flavonoid, phenylpropanoid, valine, leucine and isoleucine, steroid, carotenoid and terpenoid biosynthesis, as described in KEGG database (Figure 3). Abundance of these metabolites varied greatly between diverse sorghum accessions.

### 3.3. Grain Metabolome of Temperate and Tropical Sorghums

**Figure 4:**
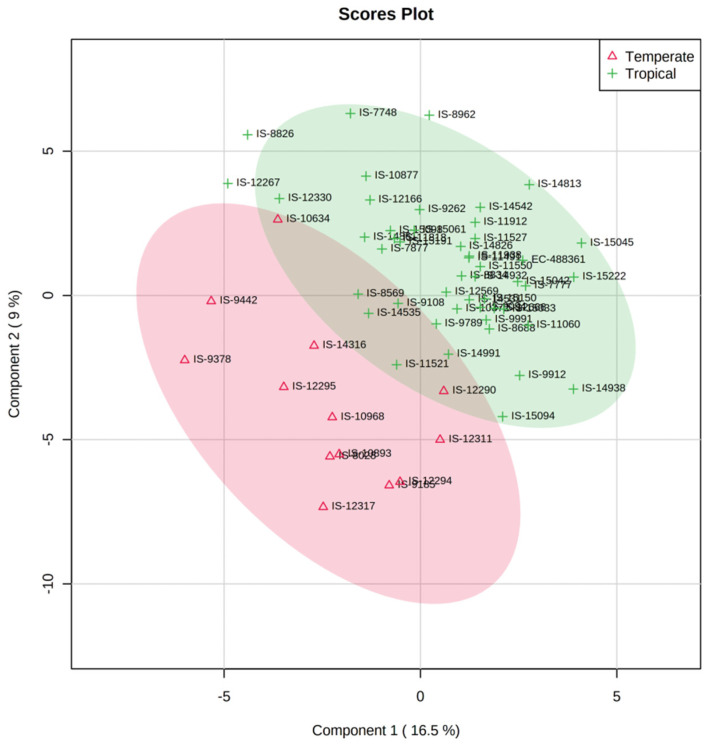
Partial least squares discriminant analyses (PLS-DA) analysis of sorghum grain metabolome. The first two principal components (PCs) explain 25.5% of variance separating tropical and temperate sorghum.

To understand the grain metabolome differences between the tropical and temperate sorghums, metabolite content of 13 temperate sorghum lines was compared against 48 tropical sorghum lines. PLS-DA analysis revealed that the first two components separated the temperate and tropical sorghum with limited overlaps (Figure 4). Distinct grouping of sorghum lines based on their grain metabolome suggests that tropical and temperate sorghum may have entirely different metabolic machinery for their adaptation to their environments.

**Figure 5:**
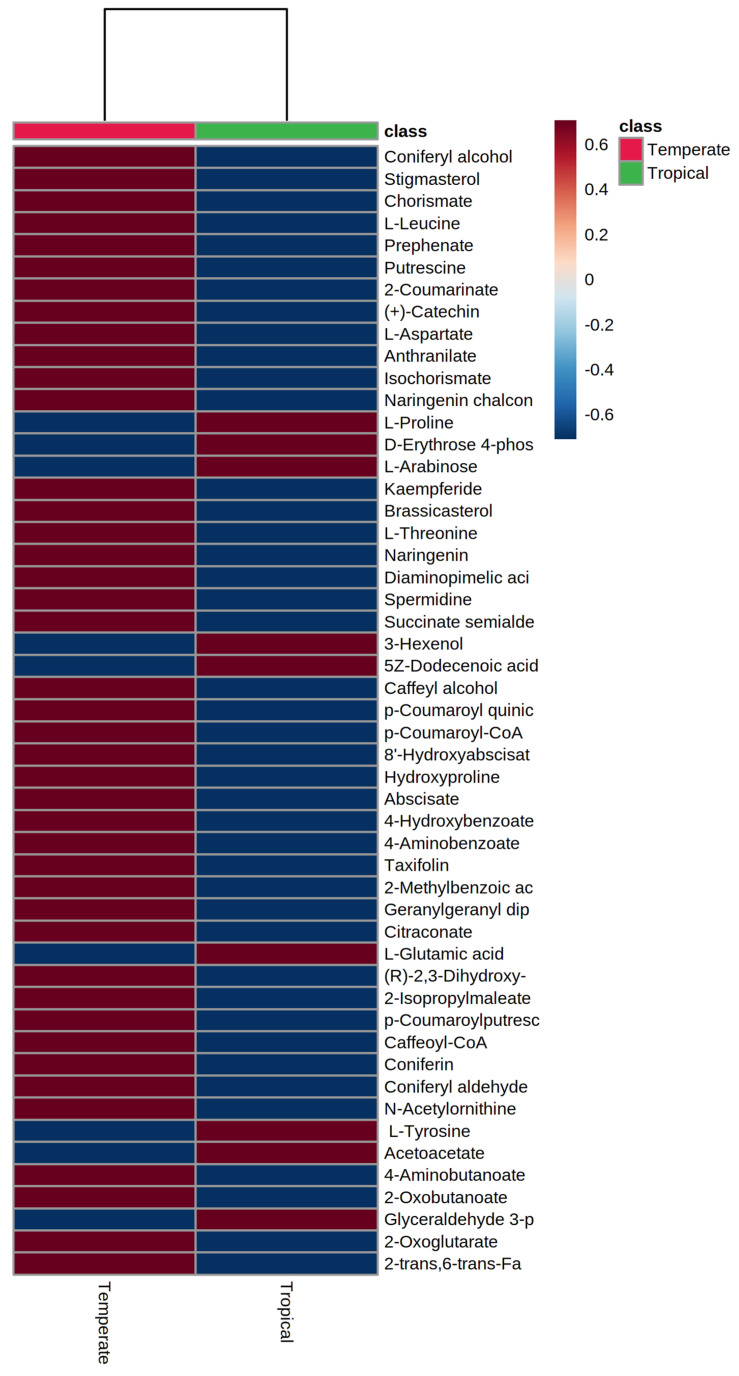
Heatmap of PLS-DA VIP metabolites differentiating temperate and tropical sorghum.

To differentiate the tropical and temperate sorghums based on their grain metabolome, PLS-DA analysis and heat map analysis was performed, which revealed 51 variable importance in projection (VIP) metabolites significantly varying between the tropical and temperate sorghums (Figure 5), including eight phenylpropanoids, five flavonoids and two sterols. This analysis identified 42 metabolites higher in temperate and 9 metabolites higher in tropical sorghums. It was observed that phenylpropanoids, sterols, amino acids and flavonoids can be used to differentiate between the tropical and temperate sorghum. Temperate sorghum grains were found to have increased levels of phenylpropanoids, flavonoids, steroids and amino acids (Figure 5). In contrast, tropical sorghums had higher levels of L-Proline, L-Glutamic acids, L-Arabinose, D-Erythrose 4 P and a few other carbohydrates and fatty acids.

### 3.4. Hierarchical Clustering

**Figure 6:**
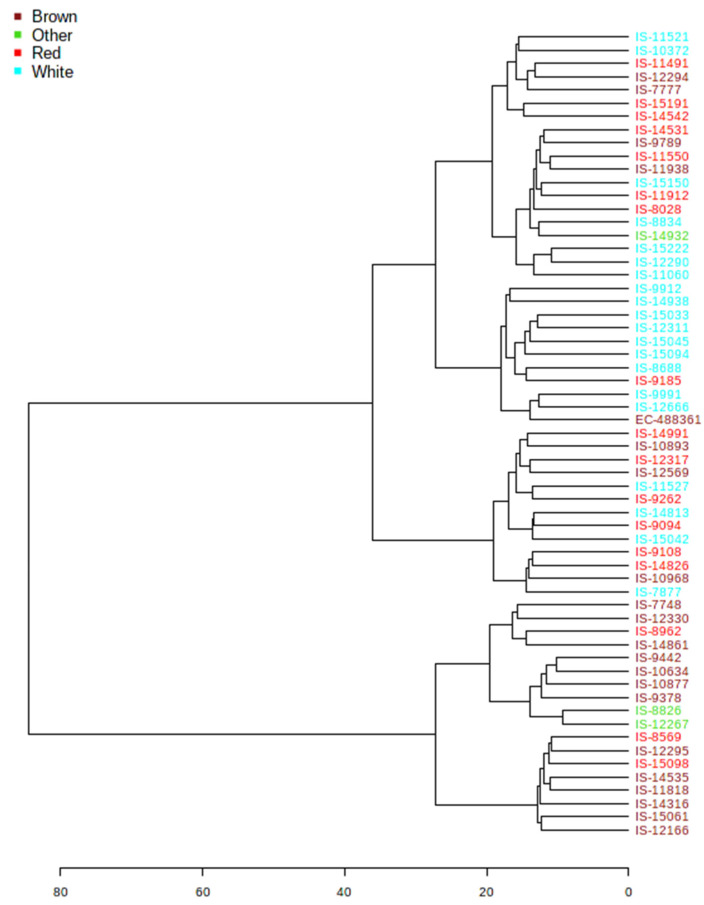
Hierarchical clustering of sorghum accessions based on its metabolite profiles.

Hierarchical clustering was performed to understand the metabolic diversity among the 61 diverse sorghum accessions and grouped into two major clusters with 18 accessions possessing brown and red pericarps (164A, 165A, 166A and 166B) and the second cluster of 43 accessions predominantly possessing light red (164B, 165B) and white pericarps (Figure 6).

### 3.5. Multivariate and Univariate Analyses

**Figure 7:**
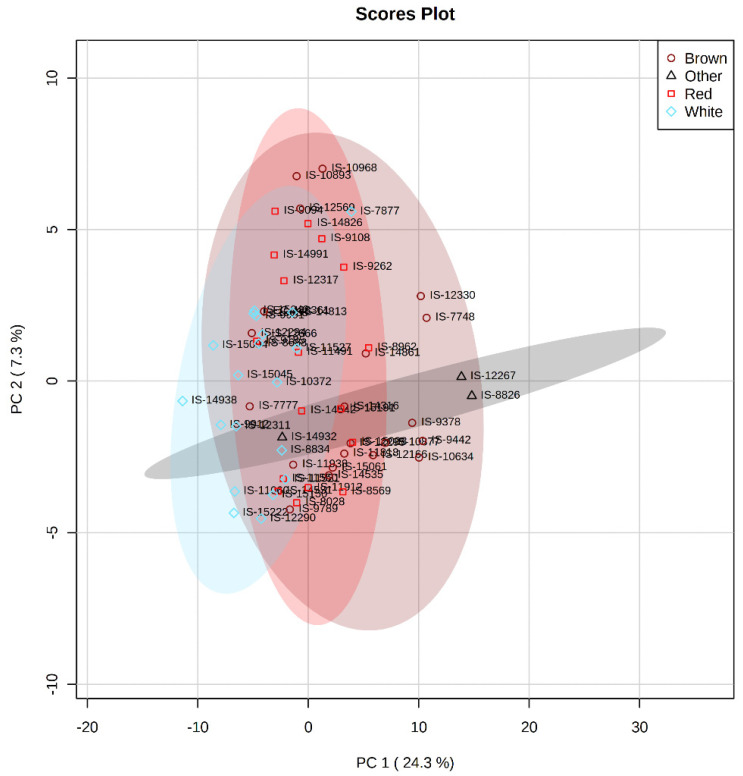
Genetic variation for accumulation of metabolites among diverse sorghum accessions obtained through principal component analysis.

**Figure 8:**
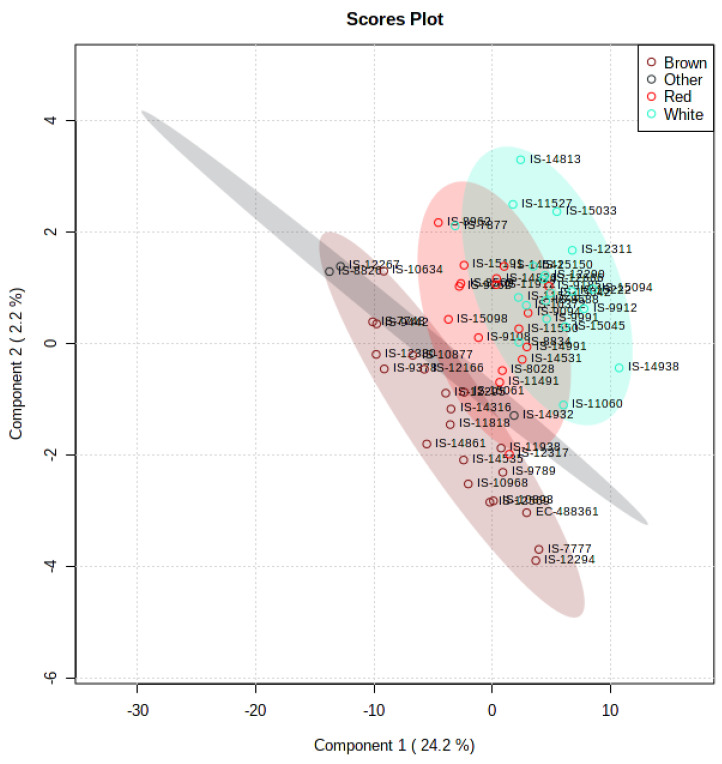
Score plots of partial least square-discriminant analysis (PLS-DA) using the data generated through non-targeted metabolomics analysis of diverse sorghum genotypes differing in grain color.

**Figure 9:**
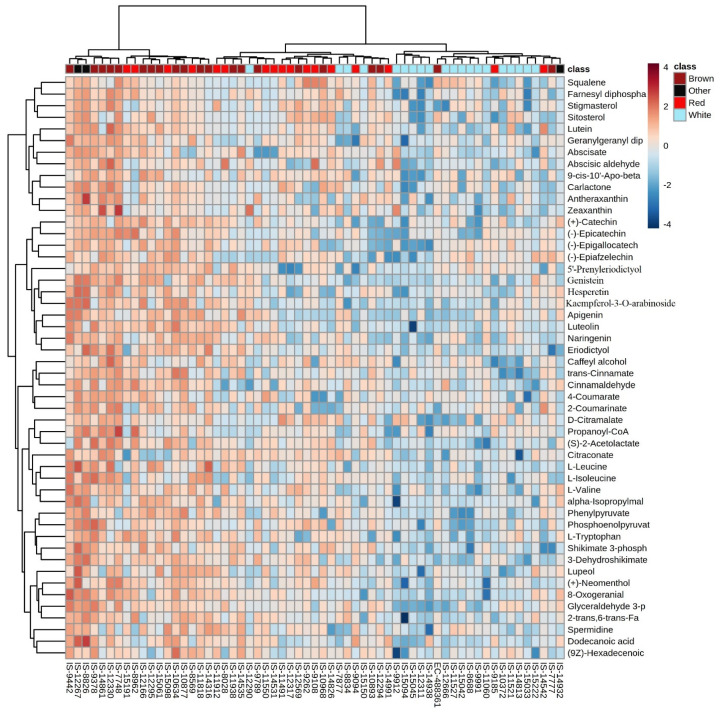
Sixty-one sorghum genotypes were clustered based on the accumulation pattern of 50 metabolites identified based on VIP scores from PLS-DA model. Color of the cell indicated the relative abundance of metabolites as indicated in the side color scale. Columns represent genotypes and rows represent a metabolite. Color of cells in the header row indicates the grain color category.

Multivariate analysis such as PCA and PLS-DA was performed to measure the genetic variation for accumulation of secondary metabolites. PCA in the 61 diverse sorghum accessions provides a preliminary estimate of the overall metabolic differences between different grain color groups and the degree of variability between samples within the group. PCA revealed that the first two components, PC1 (24.3%) and PC2 (7.3%), explain a cumulative variance of 31.6% (Figure 7). To group the diverse sorghum genotypes differing in grain color using the grain metabolome data, PLS-DA analysis was performed according to the first two components. PLS-DA model explained a cumulative variance of 26.4% (Figure 8), which identified 94 metabolites having VIP score of more than 1, indicating that these metabolites may be responsible for the metabolic variation between different colored sorghum grains (Table 1). Among the 94 VIP scored metabolites, 29 metabolites were flavonoids, followed by carboxylic acids (15 metabolites) and phenylpropanoids (13 metabolites). Four different uncommon “condensed tannins” such as catechins, epicatechins, epigallocatechins and epiafzelechin were detected in a few brown and black sorghum accessions. Heat map showing the abundance or expression levels of the top 50 metabolites having VIP score >1 is shown as described in Figure 9. Among the 94 metabolites with high VIP scores, flavonoids, phenylpropanoids and steroids contributed significantly to PC1, and they were abundant in red, brown and black grain sorghums.

Univariate analysis of variance (one-way ANOVA) was performed for identifying significant metabolites differing between different colored grain sorghum accessions. Among the 221 metabolites detected, 89 metabolites were significantly different between the accessions (Table 1). Results of one-way ANOVA were similar to the results of PLS-DA analysis.

### 3.6. Fold Change Analysis

**Figure 10:**
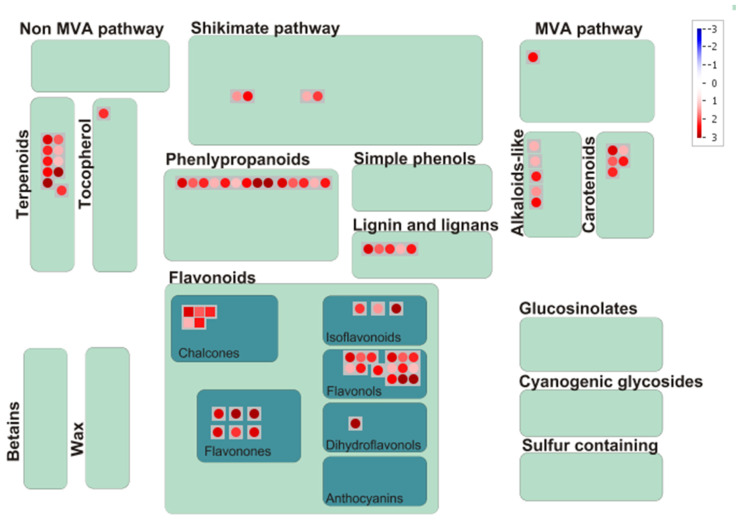
Pathway mapping of secondary metabolites up-regulated in the colored sorghum grains over the white grains.

Abundance of the metabolites in the colored grains (red, brown, yellow and black) was compared against their respective abundance in the white grains, which identified 161 up-regulated, 2 down-regulated and 58 unchanged metabolites in colored sorghum grains when compared to white grains (Supplementary Table S3; Figure 10). Pattern of accumulation and changes in the abundance of metabolites indicated that secondary metabolites were relatively more abundant in colored sorghum grains than in the white grain sorghum. Metabolites involved in the mevanolate and non-mevanolate pathways did not show any significant difference between white and colored sorghum types. White and colored sorghum grains did not differ significantly in their glucosinolates, cyanogenic glycosides and phenolics (Figure 10). Metabolites belonging to terpenoids, phenylpropanoids, flavonoids, lignins, alkaloids and carotenoids showed significant difference in their accumulation between the white and colored sorghum grains. Overall, colored sorghum grains were found to contain significantly elevated levels of these health-benefiting secondary metabolites (Figure 10).

### 3.7. Mapping of Significant Metabolites onto Metabolic Pathways

**Figure 11:**
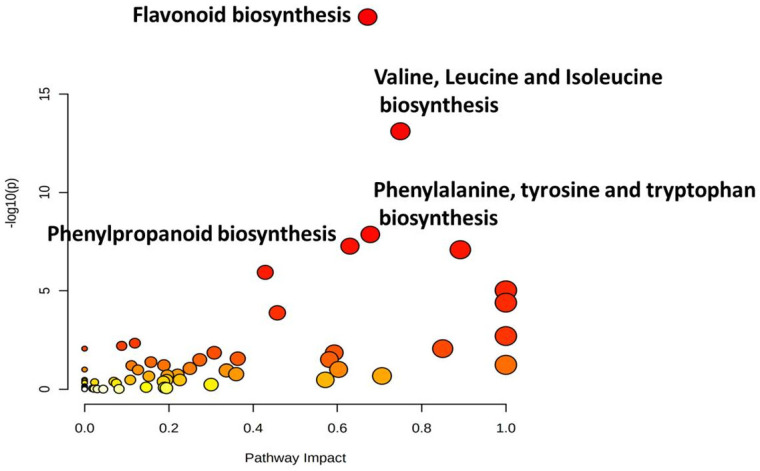
Major pathways contributing towards diversification of grain color in sorghum.

Pathway mapping using KEGG database identified 13 significant metabolic pathways showing FDR value ≤ 0.05 (Table 2; Figure 11). Flavonoid pathway showed the highest −log (*p*) value of 18.9 followed by valine, leucine and isoleucine biosynthesis (13.1); phenylalanine, tyrosine and tryptophan biosynthesis (7.9); and phenylpropanoid biosynthesis (7.3).

## 4. Discussion

Recent advancements in food technology have caused sorghum grain to become one of the major ingredients in the food industry due to its gluten-free nature. Hence, they serve as an alternate food for patients with diabetes, cardiovascular problems, obesity, immunological disorders and celiac disease [28,29,48]. Speciality grain sorghum is reported to contain bioactive compounds such as phenolic compounds, including phenolic acids (benzoic and cinnamic acids), flavonoids (3-deoxyanthocyanidins), condensed tannins (proanthocyanidins, flavin-3-ols), lignin and stilbenes produced through phenylpropanoid pathway [9,16] possessing anti-microbial activity, and anti-inflammatory and anticancer activities [49,50]. Several in-vitro studies have reported on the strong anti-oxidative and anti-inflammatory capacity of sorghum compounds [51,52]. According to the phenolic profile and color, sorghum is broadly classified into five types: white, black, brown, red and yellow [40]. Black sorghum is reported to have the highest total phenolic contents when compared to other colored (brown, red, yellow and white) sorghums [11]. However, a large number of conserved and widely diversified sorghum germplasms are underutilized. This is mainly due to lack of information on the genetic diversity and genetic basis of metabolic traits of economic importance.

Several studies have reported nutritional benefits (e.g., Fe, Zn and carotenoids) of sorghum [53,54,55], but limited attempts have been made to measure the genetic diversity of sorghum for its secondary metabolite accumulation and therapeutic properties. Plant metabolomics may help us to dissect the relationship between biological processes and phenotypes as well as their nutritive potential [56]. Non-targeted metabolomics is applied in various crops to measure genetic variation for nutritional/therapeutic traits and thus allow researchers to achieve genetic improvement of specific metabolites/biomarkers [32,33,36,57]. The present study was performed to measure genetic variation for accumulation of secondary metabolites in a diverse set of sorghum germplasm lines through non-targeted metabolomics using GC-MS/MS, paving the way for breeding fortified sorghum varieties.

Inclusion of sorghum accessions differing in grain color and growth habitat helped to assess the inter relationship with metabolite accumulation. Understanding the pattern of secondary metabolites accumulation helped to identify molecular factors underlying plant adaptation to diverse environments and nutritional/health benefits. GC-MS/MS analysis of grain metabolome in 61 diverse sorghum accessions identified a total of 221 known metabolites (Supplementary Table S2). These 221 metabolites were from different classes of primary and secondary metabolites (Figure 2) with significant role in 27 sub-pathways listed in KEGG database (Figure 3). The accessions were with clear distribution for carboxylic acids (50), flavonoids (35), amino acids (25), phenylpropanoids (21) and sesquiterpenoids (10) and were predominant in sorghum metabolome.

Metabolite profiling enabled one to understand the metabolic basis of accessions adaptation to widely varying environments. First two components of PLS-DA distinguished tropical and temperate sorghum accessions with minimum overlaps (Figure 4). Clustering based on grain metabolome revealed that these accessions possess metabolic machineries for adaptation to their environments. It was noticed that relative abundance of 51 different metabolites was significantly different between tropical and temperate sorghum lines based on PLS-DA (Figure 5). Different metabolites of amino acids, phenylpropanoids, flavonoids and a few sugars can be used to differentiate between the tropical and temperate sorghum (Figure 5). In general, temperate accessions possessed higher levels of metabolites such as phenylpropanoids, amino acids, flavonoids and steroids, whereas tropical accessions were rich in carbohydrate metabolites and stress-related amino acids. Tropical sorghums from Cameroon, Chad, Ethiopia, Kenya, Nigeria, Sudan, Uganda and Israel accumulated significantly higher levels of nine metabolites, including L-proline, L-arabinose, L-glutamic acid and glyceraldehyde 3 phosphate (Figure 5). Similar observations were reported in rice [32], where stress-tolerance-related metabolites were identified in the grains of tropical *indica* when compared to temperate *japonica* rice grains. PCA and PLS-DA analyses on grain metabolome showed that the metabolite compositions of brown and red pericarp grains are distinctly different from the white grain sorghum (Figure 7 and Figure 8).

The detected 221 different metabolites from the 61 accessions belonged to 27 classes of metabolites mapped onto 27 KEGG pathways. One-way ANOVA and PLS-DA identified 89 significant and 94 VIP metabolites, respectively. Most of the significant metabolites belonged to flavonoid (flavanones, flavones, flavan-3-ols, flavonols and dihydroflavonols), phenylpropanoids, condensed tannins and sterols, which have tremendous health benefits, including antioxidant anti-cancer properties [58,59]. Out of 221 metabolites detected, 35 were under the class of flavonoids. Heat map and fold change analysis of this study (Figure 9 and Figure 10) indicated that most of these flavonoids and other therapeutic metabolites are more abundant in dark red pericarp than in light red and white pericarp accessions. These metabolites could be used as biomarkers for discriminating diverse colored sorghum genotypes. Overall, the metabolome data generated in the grains of diverse sorghum genotypes suggests that the metabolome of colored grain sorghum are quite different from that of white sorghum.

The rare flavanones metabolites (naringenin and eriodictyol) possessing anti-cancer properties [60] were found in the brown- and red-colored sorghum accessions IS 9442, IS 10877, IS 10634 and IS 8569 (Table 3). No white-colored sorghums were found to contain significantly elevated levels of these secondary metabolites. Similar results were reported earlier in sorghum by Taylor and Awika (2017) [61]. Flavones including luteolin and apigenin were found to be abundant in the grains of brown, red and black sorghum accessions viz. IS 10634, IS 9442, IS 9378, IS 12267, IS 14535, IS 15098 and IS 11818 (Table 3). Apigenin was demonstrated to induce apoptosis of colorectal cancer cells and to activate estrogenic activities [50]. Condensed tannins, including catechin, epicatechin, epigallocatechin and epiafzelechin, detected in the grains of colored sorghum lines (IS 12166, IS 15098, IS 7748, IS 9378 and IS 14861) were reported to exhibit antioxidant, anti-inflammatory and anticancer activities [7,17,62,63]. Very interestingly, sorghum grains studied were found to accumulate nutraceutically important phytosterols viz. stigmasterol (IS 7748, IS 8826, IS 12330 and IS 12267) and sitosterol (IS 7748, IS 8826 and IS 12267) [64]. Phytosterols are functional foods known for their cholesterol-lowering ability [65]. Common dietary sources of phytosterols are vegetable oils, cereal products, vegetables and fruit. On the contrary, this study has identified sorghum lines rich in phytosterols, namely, stigmasterol and sitosterol (cholesterol-lowering ability and anti-cancer). Stigmasterols are used as a precursor for industrial production of semisynthetic progesterone, an important regulatory and tissue rebuilding hormone, as well as an intermediary in the biosynthesis of androgens, estrogens and corticoids [66]. Stigmasterol is also used as one of the precursors in the synthesis of vitamin D3 [67]. The sorghum accessions listed in Table 3 are rich in key nutraceuticals and therapeutic compounds and can be further used for trait introgression to improve adapted breeding lines.

Pathway analysis was performed to identify key metabolic pathways contributing to variation in grain color. Results revealed that grain color diversity in sorghum was attributed to flavonoid pathway (−log (*p*) = 18.923), followed by valine, leucine and isoleucine biosynthesis (−log (*p*) = 13.114), phenylalanine, tyrosine and tryptophan biosynthesis (−log(*p*) = 7.8637) and phenylpropanoid biosynthesis (−log (*p*) = 7.2676). Similar studies with three cultivars on non-targeted metabolomic analysis were conducted earlier by Xia and Wishart (2010) [46] and Zhou et al., (2020) [36]. In this study, firstly in sorghum, large-scale detailed grain antioxidants and metabolome profiling analyses using advanced bioinformatics tools in sorghum accessions diversified origins, growth habitats and grain colors were studied, with significant results.

## 5. Conclusions

The present study was aimed at unravelling metabolic signatures in the grains of a subset of 61 diverse sorghum accessions differing in origin, growth habitat and grain color. GC-MS/MS analysis identified a total of 221 different metabolites mapping with a significant role in 27 sub-pathways listed in KEGG database. The results of PCA and PLS-DA analysis revealed distinguished the clustering of accessions differing in their grain color and metabolite variations. Further, metabolic signatures explained the adaptability of sorghum accessions to varied growth conditions (tropical vs. temperate). Antioxidants such as flavonoids, condensed tannins, phenolic acids, phenylpropanoids, phytosterols and amino acids were more predominant in the dark-colored (brown, black and red) sorghum grains than in the white-colored grains. Overall, this study paves the way for further genetic and molecular studies through association mapping and omics approaches to identify genetic factors determining the accumulation of nutraceuticals and therapeutically important metabolites in sorghum. Further, identified genetic stocks of sorghum rich in nutraceutical compounds may be utilized in food fortification and bio-fortification programs.

## Figures and Tables

**Table 1:** List of significant metabolites differentiating diverse sorghum accessions.

S. No.	Compound	Class	PLS-DA VIP Score	ANOVA *p* Value
1	Naringenin	Flavonoids	2.1346	9.53 × 10^−9^
2	(+)-Catechin	Flavonoids	1.9378	9.88 × 10^−7^
3	Cinnamaldehyde	Phenylpropanoids	1.8646	1.39 × 10^−6^
4	Stigmasterol	Steroids	1.8592	3.71 × 10^−6^
5	(−)Epicatechin	Flavonoids	1.7997	1.25 × 10^−5^
6	alpha-Isopropylmalate	Carboxylic acids	1.7905	0.00013398
7	trans-Cinnamate	Phenylpropanoids	1.7703	0.00015462
8	L-Leucine	Amino acids	1.755	0.00017873
9	Luteolin	Flavonoids	1.7147	1.82 × 10^−5^
10	Sitosterol	Steroids	1.607	0.00022826
11	Antheraxanthin	Carotenoids	1.6026	0.00039374
12	L-Valine	Amino acids	1.5648	0.00047442
13	Apigenin	Flavonoids	1.5496	1.54 × 10^−7^
14	Caffeyl alcohol	Phenylpropanoids	1.5412	0.00017893
15	5′-Prenyleriodictyol	Flavonoids	1.5036	7.06 × 10^−6^
16	Kaempferol-3-O-arabinoside	Flavonoids	1.4855	1.57 × 10^−5^
17	Genistein	Flavonoids	1.4852	0.00021481
18	Coniferyl aldehyde	Phenylpropanoids	1.4738	0.00034236
19	Eriodictyol	Flavonoids	1.4696	7.06 × 10^−6^
20	Hesperetin	Flavonoids	1.4548	5.14 × 10^−5^
21	(−)-Epigallocatechin	Flavonoids	1.4533	9.29 × 10^−5^
22	Abscisate	Apocarotenoids	1.4504	0.00072293
23	2-Coumarinate	Phenylpropanoids	1.4217	0.00079068
24	Lutein	Carotenoids	1.4109	0.0012749
25	Abscisic aldehyde	Sesquiterpenoids	1.3993	0.0021929
26	L-Isoleucine	Amino acids	1.3974	0.00065042
27	Phosphoenolpyruvate	Carboxylic acids	1.3583	0.00078092
28	Shikimate 3-phosphate	Carboxylic acids	1.355	0.00098977
29	Propanoyl-CoA	Fatty acids	1.3536	0.0012642
30	L-Tryptophan	Amino acids	1.3512	0.0019882
31	4-Coumarate	Phenylpropanoids	1.3482	0.0013115
32	Lupeol	Triterpenoids	1.3391	0.0013742
33	Geranylgeranyl diphosphate	Diterpenoids	1.3276	0.00068171
34	Farnesyl diphosphate	Sesquiterpenoids	1.3202	0.0024475
35	Phenylpyruvate	Carboxylic acids	1.3156	0.0026778
36	Dodecanoic acid	Fatty acids	1.3156	0.0031825
37	Squalene	Triterpenoids	1.314	0.0023789
38	D-Citramalate	Carboxylic acids	1.3084	0.0026724
39	(9Z)-Hexadecenoic acid	Fatty acids	1.3059	0.0030008
40	(+)-Neomenthol	Monoterpenoids	1.3013	Nil
41	(S)-2-Acetolactate	Carboxylic acids	1.2963	0.0021652
42	2-trans,6-trans-Farnesal	Sesquiterpenoids	1.2927	0.0022391
43	8-Oxogeranial	Monoterpenoids	1.2855	0.0024556
44	Zeaxanthin	Carotenoids	1.2822	0.0034374
45	Citraconate	Carboxylic acids	1.268	Nil
46	Glyceraldehyde 3-phosphate	Organophosphate	1.2647	0.019693
47	Carlactone	Lactones	1.2632	0.0048748
48	Spermidine	Amino acids	1.2617	0.0029642
49	(−)-Epiafzelechin	Flavonoids	1.254	0.00313
50	3-Dehydroshikimate	Carboxylic acids	1.2521	0.0066344
51	Naringin	Flavonoids	1.2499	0.0072895
52	Eriocitrin	Flavonoids	1.2455	0.0078144
53	Brassicasterol	Steroids	1.2425	0.0095876
54	4-Coumaryl alcohol	Phenylpropanoids	1.2412	0.010089
55	9-cis-10′-Apo-beta-carotenal	Apocarotenoids	1.2407	0.011766
56	Campesterol	Steroids	1.2378	0.012192
57	Dihydrokaempferol	Flavonoids	1.2158	0.013448
58	Pentahydroxyflavanone	Flavonoids	1.2083	0.0040772
59	(S)-2-Aceto-2-hydroxybutanoate	Carboxylic acids	1.2022	0.0052647
60	Kaempferol	Flavonoids	1.2011	0.0067844
61	Kaempferide	Flavonoids	1.2	0.0042311
62	Caffeic aldehyde	Phenylpropanoids	1.1981	0.0075665
63	p-Coumaraldehyde	Phenylpropanoids	1.1949	0.0099842
64	(R)-2,3-Dihydroxy-3-methylpentanoate	Carboxylic acids	1.1919	0.011751
65	8′-Hydroxyabscisate	Carboxylic acids	1.1914	0.0042353
66	2-Oxoisocaproate	Carboxylic acids	1.1623	0.0075086
67	beta-D-Glucopyranosyl abscisate	Carboxylic acids	1.1606	0.0099713
68	9′-cis-Neoxanthin	Carotenoids	1.1532	0.00491
69	Presqualene diphosphate	Triterpenoids	1.1516	0.0094175
70	Ferulate	Phenylpropanoids	1.1463	0.010938
71	L-Phenylalanine	Amino acids	1.1446	0.012012
72	1-Deoxy-D-xylulose 5-phosphate	Sugar Phosphates	1.1438	0.003749
73	beta-Tocopherol	Prenol lipids	1.1416	Nil
74	Luteolin 7-glucoside	Flavonoids	1.1386	0.012662
75	2′,5-Dimethoxyflavone	Flavonoids	1.1308	0.012898
76	Eriodictyol-7-O-glucoside	Flavonoids	1.1293	0.013856
77	Homoeriodictyol	Flavonoids	1.1217	0.01464
78	Naringenin chalcone	Flavonoids	1.1173	0.0147
79	Indoleglycerol phosphate	Sugar Phosphates	1.1098	Nil
80	Taxifolin	Flavonoids	1.1027	0.017069
81	(S)-3-Methyl-2-oxopentanoate	Carboxylic acids	1.0996	0.0040564
82	Fustin	Flavonoids	1.0924	0.015207
83	Galangin	Flavonoids	1.0867	0.015721
84	Phloretin	Flavonoids	1.0832	0.016724
85	Garbanzol	Flavonoids	1.0786	0.016737
86	Apigenin-7-O-glucoside	Flavonoids	1.0737	0.017055
87	(S)-2,3-Epoxysqualene	Triterpenoids	1.0709	Nil
88	Chorismate	Carboxylic acids	1.0667	0.020117
89	2-C-Methyl-D-erythritol 4-phosphate	Fatty Alcohols	1.0543	0.019126
90	5-Hydroxyconiferaldehyde	Phenylpropanoids	1.0501	0.017298
91	Caffeoyl-CoA	Phenylpropanoids	1.0492	0.017933
92	p-Coumaroyl-CoA	Phenylpropanoids	1.0328	0.01822
93	L-Tyrosine	Amino acids	1.0116	0.01873
94	Demethylphylloquinol	Prenol lipids	1.0104	0.01881

PLS-DA, partial least square-discriminant analysis; VIP, variable importance in projections; ANOVA, analysis of variance; *p*, probability level.

**Table 2:** List of metabolic pathways significantly contributing to grain color diversity.

S. No.	Pathway Name	Raw *p*	−log(*p*)	FDR
1	Flavonoid biosynthesis	1.19 × 10^−19^	18.923	1.15 × 10^−17^
2	Valine, leucine and isoleucine biosynthesis	7.69 × 10^−14^	13.114	3.69 × 10^−12^
3	Phenylalanine, tyrosine and tryptophan biosynthesis	1.37 × 10^−8^	7.8637	4.38 × 10^−7^
4	Phenylpropanoid biosynthesis	5.40 × 10^−8^	7.2676	1.30 × 10^−6^
5	Tyrosine metabolism	8.19 × 10^−8^	7.087	1.57 × 10^−6^
6	Ubiquinone and other terpenoid-quinone biosynthesis	1.16 × 10^−6^	5.9359	1.85 × 10^−5^
7	C5-Branched dibasic acid metabolism	9.51 × 10^−6^	5.0218	0.000114
8	Isoquinoline alkaloid biosynthesis	9.51 × 10^−6^	5.0218	0.000114
9	Sesquiterpenoid and triterpenoid biosynthesis	4.03 × 10^−5^	4.3947	0.00043
10	Arginine and proline metabolism	0.000133	3.8767	0.001275
11	Biosynthesis of secondary metabolites - unclassified	0.002016	2.6955	0.017596
12	Valine, leucine and isoleucine degradation	0.004638	2.3336	0.037106
13	Carotenoid biosynthesis	0.006374	2.1956	0.047071

FDR, false discovery rate is a measure of error; −log(*p*) represents significance at probability (*p*) ≤ 0.05.

**Table 3:** List of accessions enriched with nutraceutical and therapeutic metabolites and their potential applications.

Scheme	Class	Metabolites	Enriched Sorghum Accessions	Uses	References
1	Squalene	Triterpenoids	IS 7748, IS 9108, IS 9262, IS 10968	Anti-cancer, anti-bacterial and cholesterol-lowering ability	[68,69,70]
2	Stigmasterol	Steroids	IS 7748, IS 8826, IS 12330, IS 12267	Anti-cancer and cholesterol-lowering ability, reduces risk of cardiovascular diseases	[71,72,73]
3	Sitosterol	Steroids	IS 7748, IS 8826, IS 12267	Prevention of cervical cancer, lowers cholesterol level	[74]
4	Lutein	Carotenoids	IS 9378, IS 12330, IS 14542	Delays/inhibits age-related macular degeneration, improves cardiovascular health, and anti-cancer	[75,76]
5	Zeaxanthin	Carotenoids	IS 14861, IS 12290, IS 7748	Protective factor in age-related macular degeneration (AMD), reduces diabetic retinopathy and inhibits cataract growth	[77,78,79]
6	(+)-Catechin	Flavonoids	IS 12166, IS 15098, IS 7748, IS 9378, IS 14861	Prevents/reduces skin damage; antioxidant; anti-inflammatory; anti-viral; and anti-cancer	[80]
7	(−)-Epicatechin	Flavonoids	IS 15191, IS 7748, IS 8962, IS 14861, IS 12330	Anti-diabetic, cytotoxic to cancer cells, antioxidant and anti-angiogenic	[81]
8	Naringenin	Flavonoids	IS 9442, IS 10877, IS 10634, IS 8569, IS 10877	Anti-cancer, cardiovascular protection, anti-viral (against hepatitis C virus) and weight control	[82]
9	Apigenin	Flavonoids	IS 12267, IS 9442, IS 10634, IS 14535, IS 15098, IS 11818	Anti-cancer, activation of estrogen	[50]
10	Genistein	Flavonoids	IS 8826, IS 12267	Anti-tumor	[83]
11	(−)-Epigallocatechin	Flavonoids	IS 14316, IS 15061, IS 15098, IS 10634, IS 12330	Anti-inflammatory, anti-cancer and antioxidant	[80]
12	Hesperetin	Flavonoids	IS 8826, IS 12267	Antioxidant, lowers blood cholesterol	[84]
13	Kaempferol-3-O-arabinoside	Flavonoids	IS 9442, IS 12267, IS 12166, 1S 8826, IS 15098, IS 10634, IS 10877	Chemotherapeutic drug, antioxidant and anti-inflammatory	[85]
14	Luteolin	Flavonoids	IS 10634, IS 9442, IS 9378, IS 12267	Anti-cancer, anti-hypertensive and anti-inflammatory	[86]
15	Eriodictyol	Flavonoids	IS 12330, IS 8826, IS 9378, IS 14316	Anti-cancer, anti-inflammatory and anti- oxidant	[87]

## Data Availability

Data is contained within this article and supplementary information.

## Supplementary Materials

The following are available online at https://www.mdpi.com/article/10.3390/antiox10101511/s1. Figure S1: GC-MS/MS acquired peak data processing and annotation using MS-DIAL; Figure S2: GC-MS/MS derived chromatograms of sorghum grains differing in grain color; Table S1: Details of sorghum accessions used (grain color descriptor, origin and type); Table S2. List of metabolites identified in the grains of 61 diverse sorghum accessions; Table S3. Abundance ratio of 163 metabolites showing more than 2 fold change between colored and white grain sorghums.
