# Supplementary material for: Large-Scale Non-Targeted Metabolomics Reveals Antioxidant, Nutraceutical and Therapeutic Potentials of Sorghum
Source: Antioxidants (Basel). 2021 Sep 23;10(10):1511. doi: 10.3390/antiox10101511 (PMC8532915; doi:10.3390/antiox10101511)
Supplement: Supplementary file 1 [file antioxidants-10-01511-s001.zip › antioxidants-1381559-supplementary.pdf]

Supplementary Information

Title of the paper: "Large scale non targeted metabolomics reveals anti-oxidant, nutraceutical and therapeutic potentials of Sorghum"

Supplementary Figure S1: GC-MS/MS acquired peak data processing and annotation using MS-DIAL

Raw data from GC-MS/MS analysis

↓ Using *Reifycs* Abf Converter

Raw file converted to .abf format

↓ Ionization type: Soft, separation type : chromatography, Ion mode: Positive
MS method type : data dependent MS/MS, MS1 & MS2 : Centroid

Created an project by importing the .abf files into MS-DIAL

↓

Peak data processing and annotation using MS-DIAL

- MS1 tolerance : 0.01 Da
- MS2 tolerance : 0.025 Da
- Minimum peak height : 1000 amplitude
- Mass slice width : 0.1 Da
- Sigma window value : 0.5
- .msp files from MoNA used for annotation →

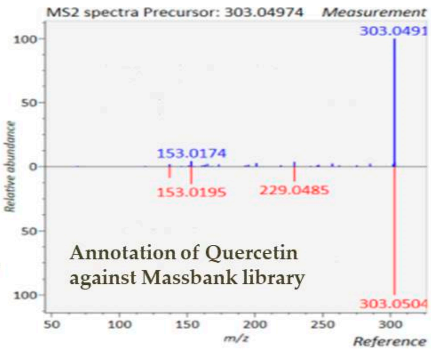

↓ Data filtering, normalization and scaling

Peak masses and its area intensities were used for further statistical analysis using MetaboAnalyst 5.0

Supplementary figure for the article entitled "Large scale non targeted metabolomics reveals anti-oxidant, nutraceutical and therapeutic potentials of Sorghum"

Supplementary Figure S2: GC-MS/MS derived chromatograms of sorghum grains differing in grain color

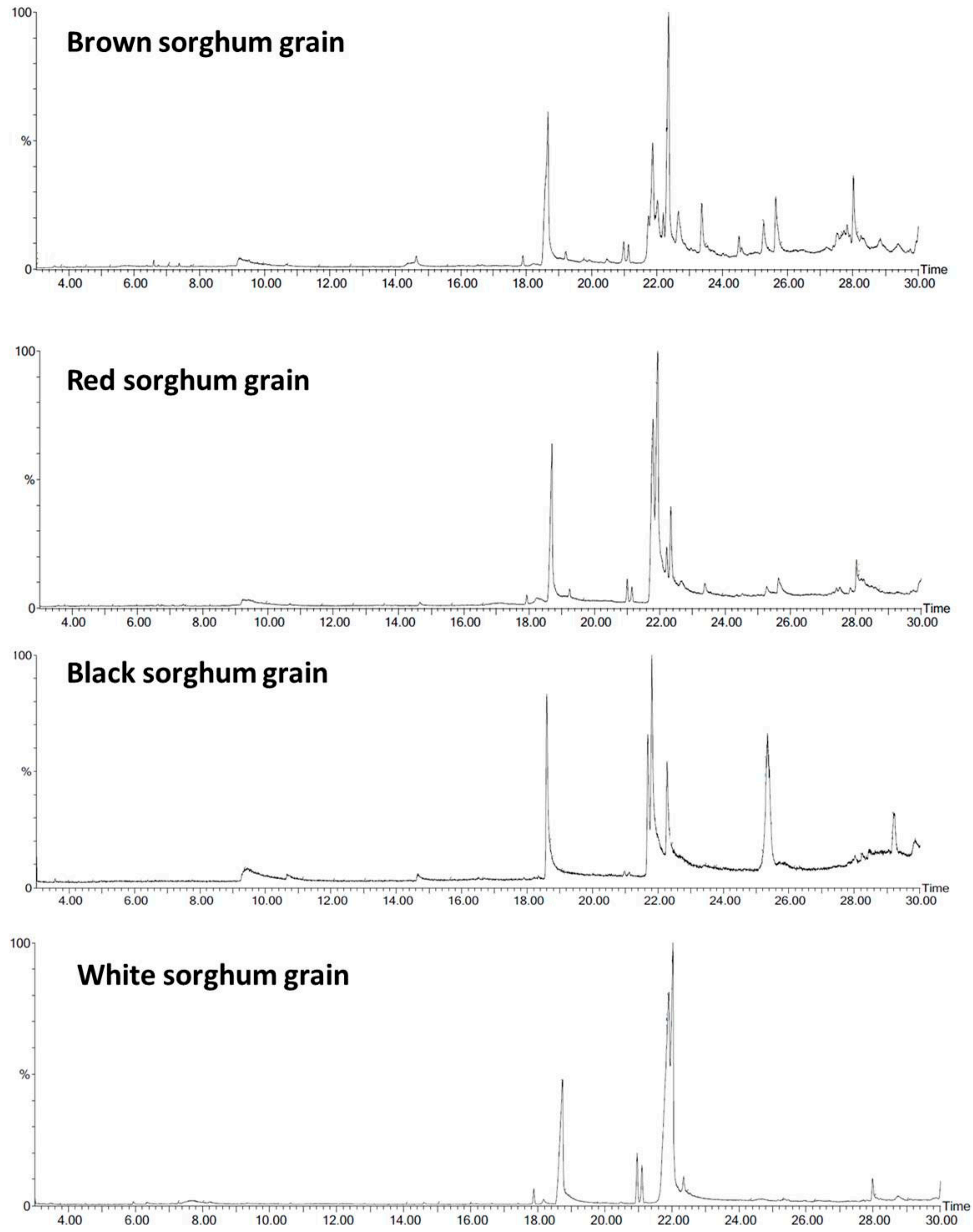

Supplementary tables for the article entitled "Large scale non targeted metabolomics reveals anti-oxidant, nutraceutical and therapeutic potentials of Sorghum"

Supplementary Table S1: Details of sorghum accessions used (grain color descriptor, origin and type)

Accessions	RHS Color grade	Color descriptor	Origin	Type
IS 7748	Greyed orange - 164A	Brown	Nigeria	Tropical
IS 7777	Greyed orange 164B	Brown	Nigeria	Tropical
IS 7877	White- 155A	White	Nigeria	Tropical
IS 8028	Greyed orange - 165B	Red	Japan	Temperate
IS 8569	Greyed orange - 165A	Red	Chad	Tropical
IS 8688	White- 155B	White	Sudan	Tropical
IS 8826	Black- 203A	Black	Kenya	Tropical
IS 8834	White- 155A	White	Kenya	Tropical
IS 8962	Greyed orange - 166B	Red	Kenya	Tropical
IS 9094	Greyed orange- 165B	Red	Kenya	Tropical
IS 9108	Greyed orange - 165B	Red	Kenya	Tropical
IS 9185	Greyed orange - 165B	Red	South Africa	Temperate
IS 9262	Greyed orange - 166B	Red	Uganda	Tropical
IS 9378	Greyed orange - 164A	Brown	South Africa	Temperate
IS 9442	Greyed orange - 164A	Brown	South Africa	Temperate
IS 9789	Greyed orange - 164B	Brown	Sudan	Tropical
IS 9912	White- 155B	White	Sudan	Tropical
IS 9991	White- 155D	White	Sudan	Tropical
IS 10372	White-155B	White	Israel	Tropical
IS 10634	Greyed orange-164A	Brown	USA	Temperate
IS 10877	Greyed orange-164A	Brown	Nigeria	Tropical
IS 10893	Greyed orange-164B	Brown	USA	Temperate
IS 10968	Greyed orange-164B	Brown	USA	Temperate
IS 11060	White- 155A	White	Ethiopia	Tropical
IS 11491	Greyed orange-165B	Red	Ethiopia	Tropical
IS 11521	White- 155A	White	Ethiopia	Tropical
IS 11527	White- 155B	White	Ethiopia	Tropical
IS 11550	Greyed orange-165B	Red	Ethiopia	Tropical
IS 11818	Greyed orange-164A	Brown	Ethiopia	Tropical
IS 11912	Greyed orange-165B	Red	Ethiopia	Tropical
IS 11938	Greyed orange-164B	Brown	Ethiopia	Tropical
IS 12166	Greyed orange-164A	Brown	Ethiopia	Tropical
IS 12267	Black- 203A	Black	Ethiopia	Tropical
IS 12290	White- 155A	White	Zimbabwe	Temperate

IS 12294	Greyed orange-164B	Brown	Zimbabwe	Temperate
IS 12295	Greyed orange-164A	Brown	Zimbabwe	Temperate
IS 12311	White- 155C	White	Zimbabwe	Temperate
IS 12317	Greyed orange-165B	Red	Zimbabwe	Temperate
IS 12330	Greyed orange-164A	Brown	Nigeria	Tropical
IS 12569	Greyed orange-164B	Brown	Sudan	Tropical
IS 12666	White- 155D	White	Ethiopia	Tropical
IS 14316	Greyed orange-164A	Brown	Swaziland	Temperate
IS 14531	Greyed orange-165B	Red	Uganda	Tropical
IS 14535	Greyed orange-164A	Brown	Kenya	Tropical
IS 14542	Greyed orange-165B	Red	Ethiopia	Tropical
EC488361	Greyed orange-164B	Brown	Ethiopia	Tropical
IS 14813	White- 155B	White	Cameroon	Tropical
IS 14826	Greyed orange-165B	Red	Cameroon	Tropical
IS 14861	Greyed orange-164A	Brown	Cameroon	Tropical
IS 14932	YELLOW- 6D	Yellow	Cameroon	Tropical
IS 14938	White- 155C	White	Cameroon	Tropical
IS 14991	Greyed orange-165B	Red	Cameroon	Tropical
IS 15033	White- 155C	White	Cameroon	Tropical
IS 15042	White- 155B	White	Cameroon	Tropical
IS 15045	White- 155B	White	Cameroon	Tropical
IS 15061	Greyed orange-164A	Brown	Cameroon	Tropical
IS 15094	White- 155C	White	Cameroon	Tropical
IS 15098	Greyed orange-166A	Red	Cameroon	Tropical
IS 15150	White- 155A	White	Cameroon	Tropical
IS 15191	Greyed orange-165A	Red	Cameroon	Tropical
IS 15222	White- 155A	White	Cameroon	Tropical

Supplementary Table S2. List of metabolites identified in the grains of 61 diverse sorghum accessions

S. no.	RT (min)	Peak m/z	Metabolite annotated	KEGG ID	Class	Pathway involved
1	9.60	134.06	L-Asparagine	C00152	Amino acids	Alanine, aspartate and glutamate metabolism
2	25.38	102.10	Succinate semialdehyde	C00232	Carboxylic acids	Alanine, aspartate and glutamate metabolism
3	8.89	145.01	2-Oxoglutarate	C00026	Carboxylic acids	Alanine, aspartate and glutamate metabolism
4	5.05	104.05	4-Aminobutanoate	C00334	Amino acids	Alanine, aspartate and glutamate metabolism
5	13.20	176.04	N-Carbamoyl-L-aspartate	C00438	Amino acids	Alanine, aspartate and glutamate metabolism
6	6.70	211.13	(-)-Jasmonic acid	C08491	Fatty acids	alpha-Linolenic acid metabolism
7	5.25	294.22	OPC-8:0	C04780	Fatty acids	alpha-Linolenic acid metabolism
8	29.27	100.09	3-Hexenol	C08492	Fatty acids	alpha-Linolenic acid metabolism
9	18.57	197.30	5Z-Dodecenoic acid	NA	Fatty acids	alpha-Linolenic acid metabolism
10	3.73	138.21	3,6-Nonadienal	C16323	Fatty acids	alpha-Linolenic acid metabolism
11	20.32	171.10	9-Oxononanoic acid	C16322	Fatty acids	alpha-Linolenic acid metabolism
12	21.70	142.20	(Z)-3-Hexen-1-ol acetate	C19757	Fatty acids	alpha-Linolenic acid metabolism
13	18.60	194.04	D-Glucuronate	C00191	Monosaccharides	Amino sugar and nucleotide sugar metabolism
14	24.47	259.19	alpha-D-Galactose 1-phosphate	C00446	Monosaccharides	Amino sugar and nucleotide sugar metabolism
15	6.81	259.07	D-Glucose 1-phosphate	C00103	Monosaccharides	Amino sugar and nucleotide sugar metabolism
16	18.21	259.19	D-Mannose 6-phosphate	C00275	Monosaccharides	Amino sugar and nucleotide sugar metabolism
17	11.32	150.05	L-Arabinose	C00259	Monosaccharides	Amino sugar and nucleotide sugar metabolism
18	15.50	145.16	Spermidine	C00315	Amino acids	Arginine and proline metabolism
19	6.42	174.22	L-Arginine	C00062	Amino acids	Arginine and proline metabolism
20	11.81	189.10	N(omega)-Hydroxyarginine	C05933	Amino acids	Arginine and proline metabolism
21	6.06	131.18	Putrescine	C00134	Amino acids	Arginine and proline metabolism

22	17.38	298.10	S-Adenosyl-L-methionine	C00019	Cofactors	Arginine and proline metabolism
23	8.82	203.22	Spermine	C00750	Amino acids	Arginine and proline metabolism
24	16.16	116.07	L-Proline	C00148	Amino acids	Arginine and proline metabolism
25	13.64	113.09	(S)-1-Pyrroline-5-carboxylate	C03912	Carboxylic acids	Arginine and proline metabolism
26	10.30	235.14	p-Coumaroylputrescine	C18326	Carboxylic acids	Arginine and proline metabolism
27	17.97	278.17	p-Coumaroylagmatine	C04498	Carboxylic acids	Arginine and proline metabolism
28	7.03	265.15	Feruloylputrescine	C10497	Carboxylic acids	Arginine and proline metabolism
29	22.20	132.00	Hydroxyproline	C01157	Amino acids	Arginine and proline metabolism
30	12.60	218.11	N-Acetyl-L-citrulline	C15532	Carboxylic acids	Arginine biosynthesis
31	9.18	175.11	N-Acetylornithine	C00437	Amino acids	Arginine biosynthesis
32	14.41	129.09	L-Glutamic acid	C00025	Amino acids	Arginine biosynthesis
33	5.11	183.05	7-Methyluric acid	C16355	Alkaloids	Caffeine metabolism
34	3.99	197.07	1,7-Dimethyluric acid	C16356	Alkaloids	Caffeine metabolism
35	7.18	183.05	1-Methyluric acid	C16359	Alkaloids	Caffeine metabolism
36	7.35	196.07	3,7-Dimethyluric acid	C16360	Alkaloids	Caffeine metabolism
37	7.77	210.00	1,3,7-Trimethylurate	C16361	Alkaloids	Caffeine metabolism
38	7.50	449.48	Geranylgeranyl diphosphate	C00353	Diterpenoids	Carotenoid biosynthesis
39	6.97	263.32	Abscisate	C06082	Apocarotenoids	Carotenoid biosynthesis
40	25.38	569.44	Zeaxanthin	C06098	Carotenoids	Carotenoid biosynthesis
41	27.36	585.43	Antheraxanthin	C08579	Carotenoids	Carotenoid biosynthesis
42	18.15	569.44	Lutein	C08601	Carotenoids	Carotenoid biosynthesis
43	12.80	500.04	9'-cis-Neoxanthin	C13431	Carotenoids	Carotenoid biosynthesis
44	4.86	248.17	Abscisic aldehyde	C13455	Sesquiterpenoids	Carotenoid biosynthesis
45	4.43	279.15	8'-Hydroxyabscisate	C15514	Carboxylic acids	Carotenoid biosynthesis
46	7.54	426.05	beta-D-Glucopyranosyl abscisate	C15970	Carboxylic acids	Carotenoid biosynthesis
47	26.56	376.27	9-cis-10'-Apo-beta-carotenal	C20692	Apocarotenoids	Carotenoid biosynthesis
48	28.44	302.13	Carlactone	C20693	Lactones	Carotenoid biosynthesis
49	18.25	250.31	Xanthoxin	C13453	Carotenoids	Carotenoid biosynthesis
50	22.95	564.43	Canthaxanthin	C08583	Carotenoids	Carotenoid biosynthesis
51	4.71	347.57	Gibberellin A1	C00859	Diterpenoids	Diterpenoid biosynthesis
52	11.34	314.94	Gibberellin A9	C11863	Diterpenoids	Diterpenoid biosynthesis
53	9.33	346.40	Gibberellin A34	C11868	Diterpenoids	Diterpenoid biosynthesis

54	3.28	347.08	Gibberellin A29	C06096	Diterpenoids	Diterpenoid biosynthesis
55	9.99	69.04	(E,E)-Geranyllinalool	C20681	Monoterpenoids	Diterpenoid biosynthesis
56	18.57	200.18	Dodecanoic acid	C02679	Fatty acids	Fatty acid biosynthesis
57	19.04	253.30	(9Z)-Hexadecenoic acid	C08362	Fatty acids	Fatty acid biosynthesis
58	17.03	317.07	3,7,4'-Tri-O-methylquercetin	C04444	Flavonoids	Flavone and flavonol biosynthesis
59	18.00	287.06	Afzelin	C16911	Flavonoids	Flavone and flavonol biosynthesis
60	18.44	151.00	Naringenin	C00509	Flavonoids	Flavonoid biosynthesis
61	12.20	107.04	Phloretin	C00774	Flavonoids	Flavonoid biosynthesis
62	21.73	287.05	Dihydrokaempferol	C00974	Flavonoids	Flavonoid biosynthesis
63	22.33	288.06	Fustin	C01378	Flavonoids	Flavonoid biosynthesis
64	25.97	271.06	Apigenin	C01477	Flavonoids	Flavonoid biosynthesis
65	26.46	285.00	Luteolin	C01514	Flavonoids	Flavonoid biosynthesis
66	28.50	305.10	Taxifolin	C01617	Flavonoids	Flavonoid biosynthesis
67	28.97	595.04	Eriocitrin	C09732	Flavonoids	Flavonoid biosynthesis
68	15.40	289.07	Eriodictyol	C05631	Flavonoids	Flavonoid biosynthesis
69	29.26	287.06	Kaempferol	C05903	Flavonoids	Flavonoid biosynthesis
70	29.48	271.05	Genistein	C06563	Flavonoids	Flavonoid biosynthesis
71	29.16	580.11	Naringin	C09789	Flavonoids	Flavonoid biosynthesis
72	23.60	417.08	Kaempferol-3-O-arabinoside	C12626	Flavonoids	Flavonoid biosynthesis
73	28.50	356.05	5'-Prenyleriodictyol	NA	Flavonoids	Flavonoid biosynthesis
74	27.16	301.10	Kaempferide	C10098	Flavonoids	Flavonoid biosynthesis
75	29.80	302.04	Pentahydroxyflavanone	C05911	Flavonoids	Flavonoid biosynthesis
76	25.44	153.01	Naringenin chalcone	C06561	Flavonoids	Flavonoid biosynthesis
77	29.26	289.07	(+)-Catechin	C06562	Flavonoids	Flavonoid biosynthesis
78	26.45	431.10	Apigenin-7-O-glucoside	C04608	Flavonoids	Flavonoid biosynthesis
79	29.54	291.08	(-)-Epicatechin	C09727	Flavonoids	Flavonoid biosynthesis
80	28.11	449.10	Luteolin 7-glucoside	C03951	Flavonoids	Flavonoid biosynthesis
81	24.13	147.00	Garbanzol	C09751	Flavonoids	Flavonoid biosynthesis
82	22.11	269.04	Galangin	C10044	Flavonoids	Flavonoid biosynthesis
83	25.86	301.07	Hesperetin	C01709	Flavonoids	Flavonoid biosynthesis
84	29.00	273.08	(-)-Epiafzelechin	C12128	Flavonoids	Flavonoid biosynthesis
85	28.12	305.06	(-)-Epigallocatechin	C12136	Flavonoids	Flavonoid biosynthesis
86	19.15	449.07	Eriodictyol-7-O-glucoside	NA	Flavonoids	Flavonoid biosynthesis
87	18.43	302.08	Homoeriodictyol	C09756	Flavonoids	Flavonoid biosynthesis
88	19.22	283.09	2',5-Dimethoxyflavone	NA	Flavonoids	Flavonoid biosynthesis
89	18.63	103.05	Pinocembrin	C09827	Flavonoids	Flavonoid biosynthesis

90	19.06	303.05	Quercetin	C00389	Flavonoids	Flavonoid biosynthesis
91	21.37	464.35	Quercetin-3-O-glucoside	C05623	Flavonoids	Flavonoid biosynthesis
92	21.70	320.05	Myricetin	C10107	Flavonoids	Flavonoid biosynthesis
93	20.09	137.07	4-Hydroxybenzoate	C00156	Carboxylic acids	Folate biosynthesis
94	24.79	360.43	Tetrahydrofolate	C00101	Cofactors	Folate biosynthesis
95	7.10	296.10	Dihydrofolate	C00415	Pterins	Folate biosynthesis
96	7.58	314.29	Dihydropteroate	C00921	Pterins	Folate biosynthesis
97	19.44	138.00	4-Aminobenzoate	C00568	Amino acids	Folate biosynthesis
98	16.62	363.22	Cyclic pyranopterin phosphate	C18239	Pterins	Folate biosynthesis
99	22.00	122.00	Thiamin diphosphate	C00068	Cofactors	Glycolysis / Gluconeogenesis
100	9.88	96.82	Glycerone phosphate	C00111	Monosaccharides	Glycolysis / Gluconeogenesis
101	21.85	237.11	L-Homoserine	C00263	Carboxylic acids	Lysine biosynthesis
102	13.64	190.10	Diaminopimelic acid	C00666	Carboxylic acids	Lysine biosynthesis
103	18.60	272.20	meso-2,6-Diaminoheptanedioate	C00680	Carboxylic acids	Lysine biosynthesis
104	13.56	134.10	L-Aspartate	C00049	Amino acids	Lysine biosynthesis
105	18.21	138.00	(+)-Neomenthol	C00553	Monoterpenoids	Monoterpenoid biosynthesis
106	16.62	166.10	8-Oxogeranial	C17622	Monoterpenoids	Monoterpenoid biosynthesis
107	24.63	169.00	Phosphoenolpyruvate	C00074	Carboxylic acids	Phenylalanine, tyrosine and tryptophan biosynthesis
108	9.86	188.13	L-Tryptophan	C00078	Amino acids	Phenylalanine, tyrosine and tryptophan biosynthesis
109	16.98	91.00	Phenylpyruvate	C00166	Carboxylic acids	Phenylalanine, tyrosine and tryptophan biosynthesis
110	17.87	173.04	3-Dehydroshikimate	C02637	Carboxylic acids	Phenylalanine, tyrosine and tryptophan biosynthesis
111	5.68	254.13	Shikimate 3-phosphate	C03175	Carboxylic acids	Phenylalanine, tyrosine and tryptophan biosynthesis
112	21.76	287.13	Indoleglycerol phosphate	C03506	Sugar Phosphates	Phenylalanine, tyrosine and tryptophan biosynthesis
113	16.89	226.10	Prephenate	C00254	Carboxylic acids	Phenylalanine, tyrosine and tryptophan biosynthesis
114	11.05	93.30	Shikimate	C00493	Carboxylic acids	Phenylalanine, tyrosine and tryptophan biosynthesis
115	9.16	196.10	3-Dehydroquinate	C00944	Carboxylic acids	Phenylalanine, tyrosine and tryptophan biosynthesis
116	16.53	96.97	D-Erythrose 4-phosphate	C00279	Sugar Phosphates	Phenylalanine, tyrosine and tryptophan biosynthesis
117	7.30	151.00	Anthranilate	C00108	Carboxylic acids	Phenylalanine, tyrosine and tryptophan biosynthesis
118	18.63	120.99	L-Phenylalanine	C00079	Amino acids	Phenylpropanoid biosynthesis
119	17.97	413.03	p-Coumaroyl-CoA	C00223	Phenylpropanoids	Phenylpropanoid biosynthesis

120	9.20	430.15	Caffeoyl-CoA	C00323	Phenylpropanoids	Phenylpropanoid biosynthesis
121	12.86	148.05	trans-Cinnamate	C00423	Phenylpropanoids	Phenylpropanoid biosynthesis
122	20.18	119.00	4-Coumarate	C00811	Phenylpropanoids	Phenylpropanoid biosynthesis
123	19.31	131.00	Cinnamaldehyde	C00903	Phenylpropanoids	Phenylpropanoid biosynthesis
124	27.22	194.00	Ferulate	C01494	Phenylpropanoids	Phenylpropanoid biosynthesis
125	10.30	131.04	4-Coumaryl alcohol	C02646	Phenylpropanoids	Phenylpropanoid biosynthesis
126	10.37	178.00	Coniferyl aldehyde	C02666	Phenylpropanoids	Phenylpropanoid biosynthesis
127	21.93	149.05	p-Coumaraldehyde	C05608	Phenylpropanoids	Phenylpropanoid biosynthesis
128	22.27	147.00	2-Coumarinate	C05838	Phenylpropanoids	Phenylpropanoid biosynthesis
129	18.15	164.08	Caffeic aldehyde	C10945	Phenylpropanoids	Phenylpropanoid biosynthesis
130	18.53	194.11	5-Hydroxyconiferaldehyde	C12204	Phenylpropanoids	Phenylpropanoid biosynthesis
131	20.15	165.05	Caffeyl alcohol	C12206	Phenylpropanoids	Phenylpropanoid biosynthesis
132	17.85	209.08	Sinapoyl aldehyde	C05610	Phenylpropanoids	Phenylpropanoid biosynthesis
133	18.22	204.00	Coniferyl alcohol	C00590	Phenylpropanoids	Phenylpropanoid biosynthesis
134	17.86	173.00	p-Coumaroyl quinic acid	C12208	Phenylpropanoids	Phenylpropanoid biosynthesis
135	11.28	196.00	5-Hydroxyconiferyl alcohol	C12205	Phenylpropanoids	Phenylpropanoid biosynthesis
136	12.42	207.00	Sinapate	C00482	Phenylpropanoids	Phenylpropanoid biosynthesis
137	12.85	73.00	Syringin	C01533	Phenylpropanoids	Phenylpropanoid biosynthesis
138	15.98	365.12	Coniferin	C00761	Phenylpropanoids	Phenylpropanoid biosynthesis
139	16.06	251.09	Sinapine	C00933	Phenylpropanoids	Phenylpropanoid biosynthesis
140	4.79	276.24	5-Amino-6-(1-D-ribitylamino)uracil	C04732	Glycosides	Riboflavin metabolism
141	4.90	184.08	L-3,4-Dihydroxybutan-2-one 4-phosphate	C15556	Sugar Phosphates	Riboflavin metabolism
142	10.95	326.30	6,7-Dimethyl-8-(D-ribityl)lumazine	C04332	Pteridines	Riboflavin metabolism
143	7.59	354.21	5-Amino-6-(5'-phosphoribosylamino)uracil	C01268	Glycosides	Riboflavin metabolism
144	21.77	455.12	Flavin adenine dinucleotide	C00016	Cofactors	Riboflavin metabolism
145	15.86	426.70	(S)-2,3-Epoxysqualene	C01054	Triterpenoids	Sesquiterpenoid and triterpenoid biosynthesis
146	14.44	426.00	Lupeol	C08628	Triterpenoids	Sesquiterpenoid and triterpenoid biosynthesis
147	17.94	204.35	Trichodiene	C01860	Sesquiterpenoids	Sesquiterpenoid and triterpenoid biosynthesis
148	19.28	204.00	Humulene	C09684	Sesquiterpenoids	Sesquiterpenoid and triterpenoid biosynthesis
149	20.77	93.01	beta-Caryophyllene	C09629	Sesquiterpenoids	Sesquiterpenoid and triterpenoid biosynthesis
150	17.85	107.00	gamma-Bisabolene	C16814	Sesquiterpenoids	Sesquiterpenoid and triterpenoid biosynthesis

151	18.44	204.34	(+)-alpha-Barbatene	C19740	Sesquiterpenoids	Sesquiterpenoid and triterpenoid biosynthesis
152	16.96	189.00	(+)-beta-Chamigrene	C19908	Sesquiterpenoids	Sesquiterpenoid and triterpenoid biosynthesis
153	14.56	204.19	(+)-Thujopsene	C20724	Sesquiterpenoids	Sesquiterpenoid and triterpenoid biosynthesis
154	16.65	363.10	Farnesyl diphosphate	C00448	Sesquiterpenoids	Steroid biosynthesis
155	15.16	411.39	Squalene	C00751	Triterpenoids	Steroid biosynthesis
156	25.95	414.39	Sitosterol	C01753	Steroids	Steroid biosynthesis
157	12.95	400.37	Campesterol	C01789	Steroids	Steroid biosynthesis
158	15.55	586.10	Presqualene diphosphate	C03428	Triterpenoids	Steroid biosynthesis
159	19.40	412.37	Stigmasterol	C05442	Steroids	Steroid biosynthesis
160	11.24	353.70	Brassicasterol	C08813	Steroids	Steroid biosynthesis
161	15.23	434.69	6-Deoxotyphasterol	C15801	Steroids	Steroid biosynthesis
162	18.79	171.15	Glyceraldehyde 3-phosphate	C00118	Organophosphate	Terpenoid backbone biosynthesis
163	20.76	91.03	2-trans,6-trans-Farnesal	C03461	Sesquiterpenoids	Terpenoid backbone biosynthesis
164	16.63	217.05	2-C-Methyl-D-erythritol 4-phosphate	C11434	Fatty Alcohols	Terpenoid backbone biosynthesis
165	18.21	214.03	1-Deoxy-D-xylulose 5-phosphate	C11437	Sugar Phosphates	Terpenoid backbone biosynthesis
166	21.85	102.02	N-Acetylserotonin	C00978	Fatty acids	Tryptophan metabolism
167	22.00	177.10	Serotonin	C00780	Alkaloids	Tryptophan metabolism
168	16.30	220.08	5-Hydroxy-L-tryptophan	C00643	Amino acids	Tryptophan metabolism
169	20.32	68.90	2-Oxoadipate	C00322	Carboxylic acids	Tryptophan metabolism
170	4.86	158.06	Indole-3-acetaldehyde	C00637	Indoles	Tryptophan metabolism
171	4.90	147.00	5-Hydroxyindoleacetaldehyde	C05634	Indoleacetic Acids	Tryptophan metabolism
172	12.60	131.00	Indole-3-acetamide	C02693	Indoleacetic Acids	Tryptophan metabolism
173	19.26	152.07	3,4-Dihydroxy-L-phenylalanine	C00355	Amino acids	Tyrosine metabolism
174	20.05	186.12	3-Fumarylpyruvate	C02514	Carboxylic acids	Tyrosine metabolism
175	21.89	473.36	4-Fumarylacetoacetate	C01061	Carboxylic acids	Tyrosine metabolism
176	27.04	198.13	4-Maleylacetoacetate	C01036	Carboxylic acids	Tyrosine metabolism
177	18.64	169.05	Homogentisate	C00544	Carboxylic acids	Tyrosine metabolism
178	27.17	92.10	3-(4-Hydroxyphenyl)pyruvate	C01179	Carboxylic acids	Tyrosine metabolism
179	27.22	137.93	Tyramine	C00483	Alkaloids	Tyrosine metabolism
180	18.25	137.05	Dopamine	C03758	Alkaloids	Tyrosine metabolism
181	20.50	88.00	Pyruvate	C00022	Carboxylic acids	Tyrosine metabolism
182	20.69	130.06	Acetoacetate	C00164	Carboxylic acids	Tyrosine metabolism

183	24.77	123.01	3,4-Dihydroxyphenylacetaldehyde	C04043	Carboxylic acids	Tyrosine metabolism
184	18.95	163.06	L-Tyrosine	C00082	Amino acids	Ubiquinone and terpenoid-quinone biosynthesis
185	28.13	227.06	Chorismate	C00251	Carboxylic acids	Ubiquinone and terpenoid-quinone biosynthesis
186	26.45	417.60	beta-Tocopherol	C14152	Prenol lipids	Ubiquinone and terpenoid-quinone biosynthesis
187	27.89	438.00	Demethylphylloquinol	C21084	Prenol lipids	Ubiquinone and terpenoid-quinone biosynthesis
188	26.38	328.08	2-Succinyl-5-enolpyruvyl-6-hydroxy-3-cyclohexene-1-carboxylate	C16519	Carboxylic acids	Ubiquinone and terpenoid-quinone biosynthesis
189	26.46	240.06	SHCHC	C05817	Carboxylic acids	Ubiquinone and terpenoid-quinone biosynthesis
190	11.19	224.17	Isochorismate	C00885	Carboxylic acids	Ubiquinone and terpenoid-quinone biosynthesis
191	21.10	456.49	Phytyl diphosphate	C05427	Diterpenoids	Ubiquinone and terpenoid-quinone biosynthesis
192	28.15	416.35	Gamma-Tocotrienol	C14155	Prenol lipids	Ubiquinone and terpenoid-quinone biosynthesis
193	28.43	310.01	Menaquinol	C05819	Prenol lipids	Ubiquinone and terpenoid-quinone biosynthesis
194	20.99	410.56	6-Geranylgeranyl-2,3-dimethylbenzene-1,4-diol	C20738	Benzenediols	Ubiquinone and terpenoid-quinone biosynthesis
195	21.81	416.37	beta-Tocotrienol	C14154	Prenol lipids	Ubiquinone and terpenoid-quinone biosynthesis
196	16.62	89.03	2-Oxoisocaproate	C00233	Carboxylic acids	Valine, leucine and isoleucine biosynthesis
197	18.22	89.05	(S)-3-Methyl-2-oxopentanoate	C00671	Carboxylic acids	Valine, leucine and isoleucine biosynthesis
198	17.05	129.02	Citraconate	C02226	Carboxylic acids	Valine, leucine and isoleucine biosynthesis
199	18.69	147.07	D-Citramalate	C02612	Carboxylic acids	Valine, leucine and isoleucine biosynthesis
200	21.11	146.17	(S)-2-Aceto-2-hydroxybutanoate	C06006	Carboxylic acids	Valine, leucine and isoleucine biosynthesis
201	18.57	148.10	D-erythro-3-Methylmalate	C06032	Carboxylic acids	Valine, leucine and isoleucine biosynthesis
202	18.06	103.06	L-Threonine	C00188	Amino acids	Valine, leucine and isoleucine biosynthesis
203	19.53	60.00	2-Oxobutanoate	C00109	Carboxylic acids	Valine, leucine and isoleucine biosynthesis
204	10.20	147.06	(R)-2,3-Dihydroxy-3-methylpentanoate	C06007	Carboxylic acids	Valine, leucine and isoleucine biosynthesis
205	14.97	177.08	alpha-Isopropylmalate	C02504	Carboxylic acids	Valine, leucine and isoleucine biosynthesis

206	14.66	175.15	3-Isopropylmalate	C04411	Carboxylic acids	Valine, leucine and isoleucine biosynthesis
207	10.68	133.07	(S)-2-Acetolactate	C06010	Carboxylic acids	Valine, leucine and isoleucine biosynthesis
208	11.11	175.06	2-Isopropylmaleate	C02631	Carboxylic acids	Valine, leucine and isoleucine biosynthesis
209	11.07	174.15	(2S)-2-Isopropyl-3-oxosuccinate	C04236	Carboxylic acids	Valine, leucine and isoleucine biosynthesis
210	13.57	422.14	Propanoyl-CoA	C00100	Fatty acids	Valine, leucine and isoleucine degradation
211	19.53	86.04	L-Leucine	C00123	Amino acids	Valine, leucine and isoleucine degradation
212	20.13	134.96	L-Valine	C00183	Amino acids	Valine, leucine and isoleucine degradation
213	19.65	130.11	L-Isoleucine	C00407	Amino acids	Valine, leucine and isoleucine degradation
214	5.35	115.07	3-Methyl-2-oxobutanoic acid	C00141	Carboxylic acids	Valine, leucine and isoleucine degradation
215	4.23	101.07	(S)-Methylmalonate semialdehyde	C06002	Carboxylic acids	Valine, leucine and isoleucine degradation
216	9.44	150.07	2-Methylbenzoic acid	C07215	Carboxylic acids	Valine, leucine and isoleucine degradation
217	13.43	104.07	(R)-3-Amino-2-methylpropanoate	C01205	Amino acids	Valine, leucine and isoleucine degradation
218	17.21	247.30	Pyridoxine phosphate	C00627	Vitamins	Vitamin B6 metabolism
219	9.52	169.07	Pyridoxine	C00314	Vitamins	Vitamin B6 metabolism
220	9.47	150.05	Pyridoxal	C00250	Vitamins	Vitamin B6 metabolism
221	12.04	184.06	4-Pyridoxylic acid	C00847	Amino acids	Vitamin B6 metabolism

Supplementary Table S3. Abundance ratio of 163 metabolites showing more than 2 fold change between colored and white grain sorghums

S.no	Compound	Fold Change	log2 (Fold Change)
1	(+)-Catechin	17.33	4.12
2	trans-Cinnamate	14.26	3.83
3	Naringenin	12.55	3.65
4	Cinnamaldehyde	10.29	3.36
5	L-Isoleucine	9.73	3.28
6	Naringenin chalcone	9.72	3.28
7	Genistein	9.45	3.24
8	Stigmasterol	9.08	3.18
9	2-Coumarinate	8.86	3.15
10	2-trans,6-trans-Farnesal	8.70	3.12
11	Apigenin	8.41	3.07
12	Eriodictyol	8.39	3.07
13	Kaempferol	8.33	3.06
14	Lutein	8.18	3.03
15	(-)-Epicatechin	8.13	3.02
16	Spermidine	7.23	2.85
17	L-Leucine	7.20	2.85
18	Presqualene diphosphate	7.10	2.83
19	Luteolin	7.07	2.82
20	Kaempferol-3-O-arabinoside	7.05	2.82
21	Sitosterol	6.84	2.77
22	Hesperetin	6.67	2.74
23	Caffeoyl-CoA	6.51	2.70
24	p-Coumaroyl-CoA	6.30	2.67
25	Campesterol	6.28	2.65
26	Abscisate	6.26	2.65
27	(S)-2,3-Epoxysqualene	6.20	2.63
28	Farnesyl diphosphate	6.11	2.61
29	(-)-Epigallocatechin	6.08	2.60
30	Squalene	6.06	2.60
31	Luteolin 7-glucoside	6.02	2.59
32	Ferulate	5.94	2.57
33	2',5-Dimethoxyflavone	5.72	2.52
34	p-Coumaraldehyde	5.54	2.47
35	Zeaxanthin	5.48	2.46

36	Dihydrokaempferol	5.48	2.45
37	Pentahydroxyflavanone	5.47	2.45
38	Caffeyl alcohol	5.41	2.44
39	Lupeol	5.34	2.42
40	Naringin	5.32	2.41
41	Eriocitrin	5.32	2.41
42	L-Tryptophan	5.32	2.41
43	5'-Prenyleriodictyol	5.31	2.41
44	Brassicasterol	5.31	2.41
45	Coniferyl aldehyde	5.29	2.40
46	Kaempferide	5.26	2.40
47	(S)-3-Methyl-2-oxopentanoate	5.15	2.37
48	4-Coumaryl alcohol	5.15	2.37
49	Caffeic aldehyde	5.12	2.36
50	Homoeriodictyol	5.07	2.34
51	Eriodictyol-7-O-glucoside	5.04	2.33
52	beta-Tocopherol	5.02	2.33
53	L-Valine	4.86	2.28
54	L-Phenylalanine	4.76	2.25
55	5-Hydroxyconiferaldehyde	4.66	2.22
56	2-Oxoisocaproate	4.64	2.22
57	Chorismate	4.64	2.21
58	(-)-Epiafzelechin	4.61	2.20
59	beta-D-Glucopyranosyl abscisate	4.58	2.20
60	Taxifolin	4.50	2.17
61	8-Oxogeranial	4.41	2.14
62	Dodecanoic acid	4.40	2.14
63	Antheraxanthin	4.38	2.13
64	Galangin	4.37	2.13
65	Phloretin	4.32	2.11
66	2-C-Methyl-D-erythritol 4-phosphate	4.31	2.11
67	(S)-2-Acetolactate	4.25	2.09
68	8'-Hydroxyabscisate	4.23	2.08
69	Abscisic aldehyde	4.22	2.08
70	4-Coumarate	4.18	2.06
71	(S)-2-Aceto-2-hydroxybutanoate	4.10	2.04
72	Demethylphylloquinol	4.08	2.03
73	(+)-Neomenthol	4.06	2.02

74	(9Z)-Hexadecenoic acid	4.04	2.01
75	Coniferyl alcohol	4.00	2.00
76	Phosphoenolpyruvate	3.99	2.00
77	Propanoyl-CoA	3.95	1.98
78	(R)-2,3-Dihydroxy-3-methylpentanoate	3.92	1.97
79	Pinocembrin	3.89	1.96
80	9-cis-10'-Apo-beta-carotenal	3.89	1.96
81	Flavin adenine dinucleotide	3.84	1.94
82	S-Adenosyl-L-methionine	3.83	1.94
83	Pyruvate	3.73	1.90
84	2-Oxoglutarate	3.71	1.89
85	L-Arginine	3.70	1.89
86	Tetrahydrofolate	3.69	1.88
87	alpha-Isopropylmalate	3.69	1.88
88	D-Glucose 1-phosphate	3.68	1.88
89	Anthranilate	3.64	1.86
90	2-Oxobutanoate	3.58	1.84
91	Glycerone phosphate	3.56	1.83
92	3-Methyl-2-oxobutanoic acid	3.50	1.81
93	L-Proline	3.50	1.81
94	L-Asparagine	3.46	1.79
95	Acetoacetate	3.46	1.79
96	L-Threonine	3.45	1.79
97	D-Glucuronate	3.43	1.78
98	Succinate semialdehyde	3.35	1.74
99	Pyridoxal	3.33	1.73
100	Prephenate	3.31	1.73
101	D-Mannose 6-phosphate	3.28	1.72
102	D-Erythrose 4-phosphate	3.28	1.71
103	Pyridoxine	3.22	1.68
104	2-Oxoadipate	3.20	1.68
105	4-Aminobutanoate	3.16	1.66
106	2-Methylbenzoic acid	3.14	1.65
107	3,4-Dihydroxy-L-phenylalanine	3.12	1.64
108	Quercetin	3.09	1.63
109	Dihydrofolate	3.04	1.60
110	Putrescine	3.03	1.60
111	N-Acetylornithine	2.98	1.58

112	N-Carbamoyl-L-aspartate	2.96	1.56
113	L-Aspartate	2.93	1.55
114	alpha-D-Galactose 1-phosphate	2.92	1.55
115	Indole-3-acetaldehyde	2.91	1.54
116	5-Hydroxy-L-tryptophan	2.90	1.54
117	L-Homoserine	2.89	1.53
118	meso-2,6-Diaminoheptanedioate	2.88	1.52
119	Spermine	2.86	1.51
120	Coniferin	2.85	1.51
121	Serotonin	2.80	1.48
122	Gibberellin A1	2.77	1.47
123	Isochorismate	2.77	1.47
124	Dihydropteroate	2.75	1.46
125	Sinapine	2.72	1.44
126	3-Dehydroquinate	2.65	1.41
127	N-Acetylserotonin	2.62	1.39
128	4-Maleylacetoacetate	2.62	1.39
129	4-Fumarylacetoacetate	2.62	1.39
130	Sinapate	2.58	1.37
131	Tyramine	2.53	1.34
132	Pyridoxine phosphate	2.52	1.33
133	1,3,7-Trimethylurate	2.51	1.33
134	1,7-Dimethyluric acid	2.49	1.32
135	(Z)-3-Hexen-1-ol acetate	2.49	1.31
136	(-)-Jasmonic acid	2.48	1.31
137	3-Hexenol	2.46	1.30
138	Canthaxanthin	2.46	1.30
139	Quercetin-3-O-glucoside	2.45	1.29
140	beta-Caryophyllene	2.41	1.27
141	5Z-Dodecenoic acid	2.39	1.26
142	3-Fumarylpyruvate	2.39	1.26
143	2-Isopropylmaleate	2.34	1.22
144	1-Methyluric acid	2.33	1.22
145	Indole-3-acetamide	2.33	1.22
146	Diaminopimelic acid	2.32	1.21
147	3-Isopropylmalate	2.31	1.21
148	3,7,4'-Tri-O-methylquercetin	2.24	1.17
149	p-Coumaroylagmatine	2.23	1.16

150	5-Amino-6-(1-D-ribitylamino)uracil	2.23	1.16
151	Homogentisate	2.22	1.15
152	4-Aminobenzoate	2.21	1.15
153	OPC-8:0	2.19	1.13
154	(S)-1-Pyrroline-5-carboxylate	2.19	1.13
155	3,4-Dihydroxyphenylacetaldehyde	2.15	1.11
156	L-Glutamic acid	2.13	1.09
157	(2S)-2-Isopropyl-3-oxosuccinate	2.10	1.07
158	6,7-Dimethyl-8-(D-ribityl)lumazine	2.06	1.04
159	6-Deoxotyphasterol	2.05	1.04
160	Hydroxyproline	2.04	1.03
161	D-Citramalate	2.01	1.01
162	Citraconate	0.40	-1.32
163	Thiamin diphosphate	0.32	-1.64
